# Sociodemographic determinants and regional disparities of first-trimester antenatal care initiation among Nigerian women: a multilevel analysis of 2018 NDHS data

**DOI:** 10.3389/fgwh.2025.1502905

**Published:** 2025-11-14

**Authors:** Zinabu Bekele Tadese, Jamilu Sani, Shimels Derso Kebede, Gemeda Wakgari Kitil, Geleta Nenko Dube, Teshome Demis Nimani

**Affiliations:** 1 Department of Health Informatics, College of Medicine and Health Science, Samara University, Semera, Ethiopia; 2 Department of Demography & Social Statistics, Federal University Birnin-Kebbi, Birnin Kebbi, Kebbi State, Nigeria; 3 Department of Health Informatics, School of Public Health, College of Medicine and Health Science, Wollo University, Dessie, Ethiopia; 4 Departments of Midwifery, College of Health Sciences, Mattu University, Mattu, Ethiopia; 5 Department of Health Informatics, College of Medicine and Health Sciences, Mattu University, Mattu, Ethiopia; 6 Department of Epidemiology and Biostatistics, School of Public Health College of Medicine and Health Science, Haramaya University, Harar, Ethiopia

**Keywords:** antenatal care, maternal health, healthcare utilization, NDHS, Nigeria

## Abstract

**Background:**

Maternal health remains a critical public health priority, particularly in low- and middle-income countries where maternal mortality rates are alarmingly high. Early antenatal care (ANC) initiation within the first trimester is essential for identifying and managing potential health risks for both mothers and their babies. Despite global efforts to promote early ANC, significant disparities persist, especially in Nigeria. This study investigates the sociodemographic determinants and regional disparities influencing the timing of ANC initiation among Nigerian women.

**Methods:**

This study utilized data from the 2018 Nigeria Demographic and Health Survey (*N*DHS), analyzing a sample of 16,542 women aged 15–49 who had given birth within five years of the survey. A multivariable multilevel logistic regression model was employed to assess the impact of individual and community-level factors on early ANC initiation. The model accounted for regional clustering to identify the most significant predictors of first-trimester ANC contact.

**Results:**

The analysis revealed that only 24.0% (*n* = 3,970) of Nigerian women-initiated ANC in the first trimester, with substantial regional disparities. The South West region had the highest prevalence (34.5%, *n* = 1,045), while the North West region had the lowest (12.5%, *n* = 609). Multivariable analysis showed that women with higher education were nearly twice as likely to initiate ANC early (AOR = 1.98, 95% CI: 1.65–2.37). Muslim women had lower odds of early ANC initiation than Catholics (AOR = 0.64, 95% CI: 0.47–0.87). Wealthier women had a significantly higher likelihood of early ANC, with the richest women being nearly three times more likely than the poorest (AOR = 2.88, 95% CI: 2.49–3.33). The final multilevel model showed a reduced intraclass correlation coefficient (ICC) of 2.6%, indicating that regional variation in ANC initiation.

**Conclusion:**

The findings highlight significant sociodemographic and regional disparities in the timing of ANC initiation among Nigerian women. To improve early ANC uptake, targeted interventions that address both individual barriers, such as education and economic status, and broader regional disparities are essential.

## Background

Even though maternal health has improved significantly over the past 20 years, there were still 287,000 maternal fatalities globally in 2020, with about 800 of those deaths being related to pregnancy and childbirth-associated preventable factors. The majority of maternal deaths (almost 95%) occurred in low-income and lower middle-income countries. Sub-Saharan Africa continues to be the region with the highest rate of maternal mortality, accounting for around 70% of all maternal deaths reported worldwide in 2020 (1). Western Africa and Central and Eastern Africa were the two regions with the greatest rates of maternal deaths from complications during and after pregnancy and delivery. During this time, the highest rates of maternal deaths were reported in South Sudan, Chad, and Nigeria (2). The majority of these deaths could have been prevented with the help of skilled medical professionals prior to, during, and after pregnancy and childbirth; however, low-resource countries lack the resources to develop or implement appropriate remedies (1–3).

Maternal health is a critical aspect of public health that significantly influences maternal and child survival rates. Antenatal care (ANC) is a vital component of maternal healthcare, providing a platform for healthcare providers to deliver essential medical, nutritional, and educational services to expectant mothers (4). ANC has long been known to be an efficient method of maximizing positive birth outcomes for expectant mothers and their unborn children. ANC is defined as the care given to an expectant mother by health care providers from the moment the pregnancy is confirmed until the commencement of labor (5, 6).

Despite global initiatives aimed at promoting early ANC contact, substantial disparities persist, especially in developing countries like Nigeria, where cultural, economic, and systemic barriers often hinder access to maternal healthcare services (7). In West Africa, a lot of pregnant women, especially teenage girls, begin their prenatal care later than necessary, which limits them access to preventive and curative services (8, 9). Nigeria has one of the highest maternal mortality rates globally, with recent estimates indicating a maternal mortality ratio (MMR) of approximately 512 deaths per 100,000 live births (10, 11). Alarmingly, some studies report even higher rates, with a recent retrospective review showing an MMR of 1,114 per 100,000 live births in a tertiary hospital setting (7, 12, 13).

Although the World Health Organization (WHO) recommends that the first ANC contact should occur within the first 12 weeks of pregnancy (6), the prevalence of first-trimester ANC initiation in Nigeria remains low, with only 24% of women beginning ANC during this critical period births (12). Recent studies have highlighted the significant influence of sociodemographic factors on the utilization of ANC services by women of reproductive age. Factors such as education, economic status, religion, age at marriage, and place of residence have been identified as key determinants of ANC utilization (14–16). Early diagnosis of pregnancy issues allows for more prompt referrals for women in high-risk categories or who have complications. The timing of the first visit is around or preferably before 16 weeks of gestational age (6). Early initiation of ANC, ideally within the first trimester, is particularly important as it facilitates the timely identification and management of potential health risks. Early ANC contact provide numerous benefits, including opportunities for early risk assessment, management of pregnancy complications, and the provision of preventive measures such as vaccinations and nutritional supplements for both the mother and foetus (10, 17–19). Thus, this study aims to explore the sociodemographic determinants of early ANC contact and regional disparities among Nigerian women. Using multivariable multilevel logistic regression analysis, this research seeks to provide a comprehensive understanding of the factors influencing early ANC visits in Nigeria. The findings of this research have the potential to contribute to the development of targeted interventions that address both individual and systemic barriers to early ANC uptake, ultimately enhancing maternal and child health outcomes.

## Methods

### Study design, setting and sampling procedure

This study employed a secondary analysis of a cross-sectional survey, using data from the 2018 Nigeria Demographic and Health Survey (NDHS) dataset. The survey is cross-sectional, nationally representative, and aims to offer demographic and health data at the national, regional, and state levels. It was conducted by the National Population Commission (NPC) with technical support provided by ICF. The NDHS data covers Nigeria's geopolitical zones, which are divided into six regions: North Central, North East, North West, South East, South, and South West. The sample for the survey was selected using a two-stage stratified cluster design sampling technique. Stratification was done by region, with 1400 enumeration areas (EAs) selected using probability proportionate to EA size in the first step, followed by 30 households selected proportionally from each EA using a systematic sampling procedure in the second stage. This resulted in the selection of 42,000 households. Information was obtained from 42,821 women aged 15–49 in the selected households using a standardized questionnaire that was administered through face-to-face interview. The questionnaire was designed to capture information on background characteristics, antenatal, delivery and postnatal care, child immunization and childhood illnesses, reproductive history and child mortality, among others. A detailed description of the sampling technique and data collection procedure used can be accessed from published material online (1).

### Source and study populations

The study's source population comprised all Nigerian women aged 15–49 who gave birth within the five years before to the survey and who had at least one antenatal care visit for their last child. Whereas. The study populations consisted of women who gave birth during the five years prior to the survey, had at least one ANC visit for their most recent child, and resided in the selected enumeration areas.

### Eligibility criteria and sample

In this study, all reproductive age women who gave birth in the five years preceding the survey and found in the selected clusters at least one night before the data collection period were included, whereas, women who had no ANC visit and unknown first date of ANC visit were excluded. Accordingly, a total of 16,542 weighted samples of reproductive age women were incorporated.

### Study variables

The outcome variable is the timing of the first antenatal care (ANC) visit. It is coded as 1 for women who initiated ANC within the first trimester of pregnancy (≤12 weeks of pregnancy) and 0 for those who started later. Early ANC contact is defined as the initiation of ANC within the first trimester (first 12 weeks of pregnancy), as recommended by the WHO guidelines (5).

The independent variables in this study encompass various sociodemographic factors, including age, education, religion, wealth index, employment status (whether the woman is currently working), and the number of births in the past five years. Additionally, community-level variables such as residence (urban or rural) and geographic region (North Central, North East, North West, South East, South, and South West) are included.

### Data management and statistical analysis

The data were extracted from the individual record (IR) file dataset and analyzed further using STATA version 17. To ensure the survey's representativeness and achieve reliable statistical estimates, the data were weighted for probability sampling and non-response using sample weights. Data cleaning, and recoding were carried out, and descriptive statistics were presented in tables. The study considered two levels of data hierarchy due to the multistage stratified cluster sampling technique used in the NDHS. The first level was the individual pregnant woman in households, and the second level comprised the enumeration areas. A multilevel mixed-effects logistic regression model was employed to identify the factors influencing the early initiation of the first trimester antenatal care services at both the individual and community levels.

Three models were developed for the multilevel logistic regression analysis to examine the contribution of different levels of factors to early ANC initiation. The first model (Model I), known as the null model, included only the region variable as a random effect to assess the extent of variation in early ANC initiation across clusters, without any explanatory variables. The second model (Model II) included only individual-level factors, excluding regional clustering, to determine their independent effects. The third model (Model III) combined both individual- and community-level factors while accounting for the regional random effect identified in the null model. Developing the models sequentially made it possible to evaluate how the inclusion of additional variables reduced unexplained regional variation and improved model fit. This hierarchical approach is consistent with previous multilevel analyses of maternal healthcare utilization based on Demographic and Health Survey data (20–22).

Both bivariable and multivariable analyses were conducted. Variables with a *P*-value of ≤0.25 in the bivariable multilevel logistic regression analysis were considered for inclusion in the multivariable analysis. In the final multivariable multilevel logistic regression, variables were considered statistically significant if they had a *P*-value of <0.05. The models were compared using Akaike's Information Criterion (AIC) and Bayesian Information Criterion (BIC), with the model having the smallest AIC and BIC selected for interpretation and inference. Random effects were used to measure the variation in early ANC initiation across clusters (enumeration areas), assessed through the Intraclass Correlation Coefficient (ICC). The analytical and statistical techniques applied in this study followed established standards for multivariate modeling as described by Hair et al. (23, 24), ensuring robust estimation and model validity.

### Ethical considerations

Ethical approval was obtained from the Demographic and Health Survey (DHS) program after completing the required request form for data access. The data used in this study were freely available, aggregated secondary data, and did not include any personal identifiers that could link back to the study participants http://www.dhsprogram.com. The requested data were used anonymously and strictly for the purposes of this study. Detailed information about the ethical considerations is provided in the NDHS 2018 report.

## Results

### Sociodemographic characteristics of the study participant

Table 1 presents the sociodemographic characteristics of the study population, consisting of 16,542 Nigerian women aged 15–49 who had given birth within five years prior to the survey. The majority of the respondents were aged between 25 and 29 years (26.45%), with a significant portion having secondary education (38.53%). Most participants identified as Muslim (54.90%), and a majority were currently working (72.60%). The wealth distribution shows that the richest category comprises 22.17% of the sample. Additionally, 52.77% of the women resided in rural areas, while the North West region had the highest representation at 29.41%. Among the women, 90.71% were married, and 53.34% had one birth in the last five years. The timing of the first antenatal care (ANC) visit revealed that 24.88% of women aged 25–29 initiated ANC within the first trimester.

**Table 1:** First trimester ANC initiation by sociodemographic characteristics of reproductive-age women in Nigeria, 2018 (*N* = 16,542).

Variables	Category	Weighted frequency	%	Timing of first ANC contact	
				Late trimester (Freq. %)	First trimester (Freq. %)
Maternal age	15–19	809	4.89	652 (80.64)	157 (19.36)
	20–24	3,104	18.76	2,369 (76.34)	734 (23.66)
	25–29	4,376	26.45	3,287 (75.12)	1,089 (24.88)
	30–34	3,780	22.85	2,823 (74.68)	957 (25.32)
	35–39	2,765	16.72	2,086 (75.44)	679 (24.56)
	40–44	1,230	7.44	936 (76.10)	294 (23.90)
	45–49	479	2.90	381 (79.51)	98 (20.49)
Educational level	No Education	5,520	33.37	4,684 (84.87)	835 (15.13)
	Primary	2,752	16.64	2,136 (77.63)	615 (22.37)
	Secondary	6,374	38.53	4,525 (70.99)	1,849 (29.01)
	Higher	1,896	11.46	1,189 (62.69)	708 (37.31)
Religion	Catholic	1,651	9.98	1,079 (65.34)	572 (34.66)
	Other Christian	5,733	34.66	3,898 (68.00)	1,834 (32.00)
	Islam	9,082	54.90	7,491 (82.49)	1,590 (17.51)
	Traditionalist	51	0.31	43 (83.43)	9 (16.57)
	Other	26	0.16	24 (91.07)	2 (8.93)
Wealth index	Poorest	2,457	14.85	2,064 (83.98)	394 (16.02)
	Poorer	3,145	19.01	2,508 (79.74)	637 (20.26)
	Middle	3,588	21.69	2,790 (77.75)	799 (22.25)
	Richer	3,685	22.28	2,805 (76.14)	879 (23.86)
	Richest	3,667	22.17	2,368 (64.57)	1,299 (35.43)
Employment status	No	4,533	27.40	3,658 (80.70)	875 (19.30)
	Yes	12,009	72.60	8,877 (73.91)	3,133 (26.09)
Births in Last 5 Years	1	8,823	53.34	6,450 (73.10)	2,373 (26.90)
	2	6,557	39.63	5,168 (78.81)	1,390 (21.19)
	3	1,077	6.5	851 (79.04)	226 (20.96)
	4 +	85	0.51	66 (77.15)	19 (22.85)
Marital status	Never In Union	407	2.46	292 (71.79)	115 (28.21)
	Married	15,005	90.71	11,418 (76.10)	3,587 (23.90)
	Living With Partner	525	3.17	381 (72.59)	144 (27.41)
	Widowed	212	1.28	165 (77.91)	47 (22.09)
	Divorced	208	1.26	162 (77.69)	46 (22.31)
	Separated	185	1.12	116 (62.65)	69 (37.35)
Residence	Urban	7,813	47.23	5,688 (72.79)	2,126 (27.21)
	Rural	8,729	52.77	6,847 (78.44)	1,882 (21.56)
Region	North Central	2,192	13.25	1,507 (68.76)	685 (31.24)
	North East	2,755	16.65	2,222 (80.64)	533 (19.36)
	North West	4,865	29.41	4,257 (87.49)	609 (12.51)
	South East	2,057	12.44	1,390 (67.58)	667 (32.42)
	South	1,647	9.96	1,177 (71.50)	469 (28.50)
	South West	3,026	18.29	1,981 (65.47)	1,045 (34.53)

### Prevalence and regional disparities of ANC at first trimester across regions

**Figure 1:**
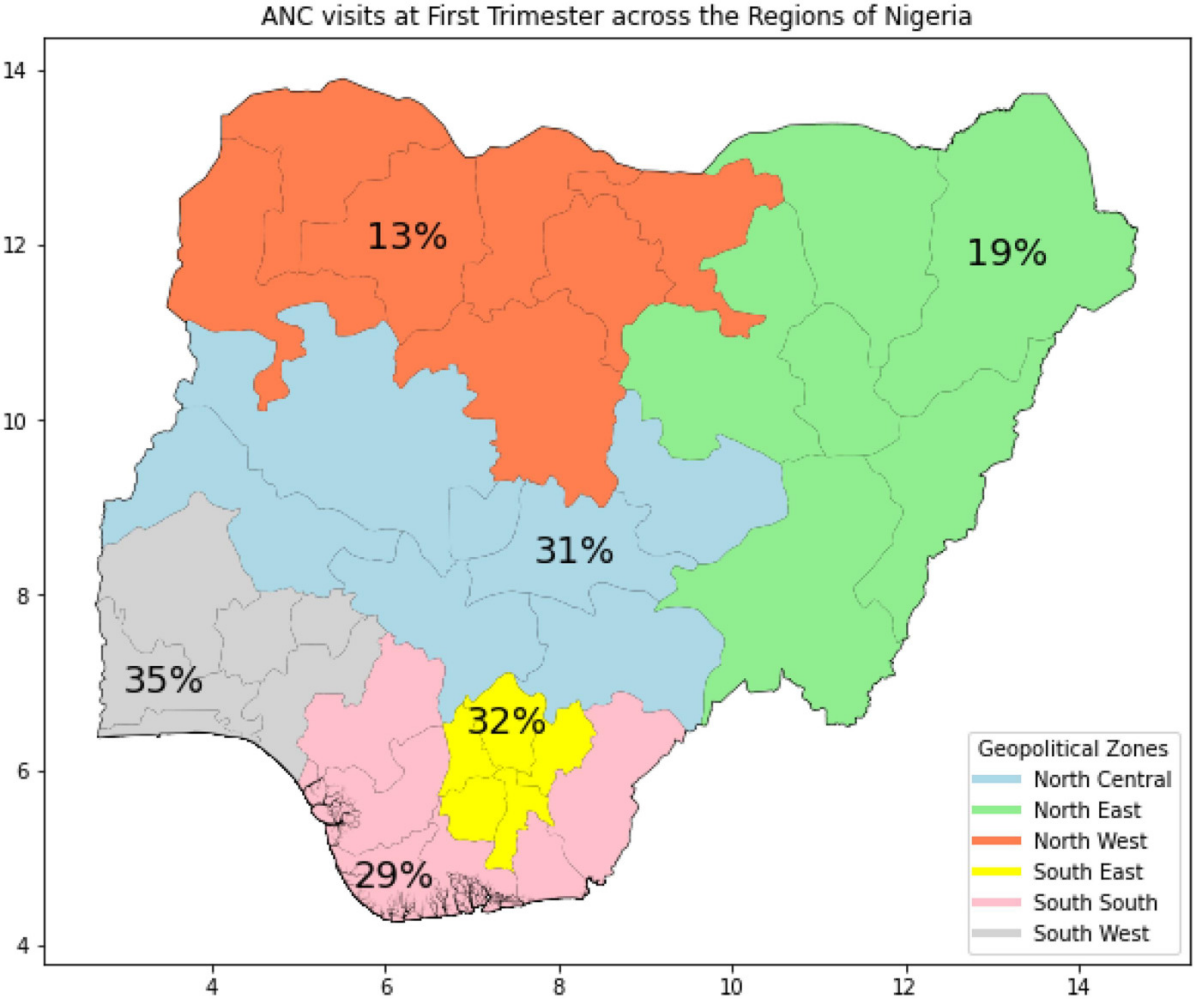
Map of Nigeria showing the regional prevalence of first ANC visit at first trimester, 2018.

**Figure 2:**
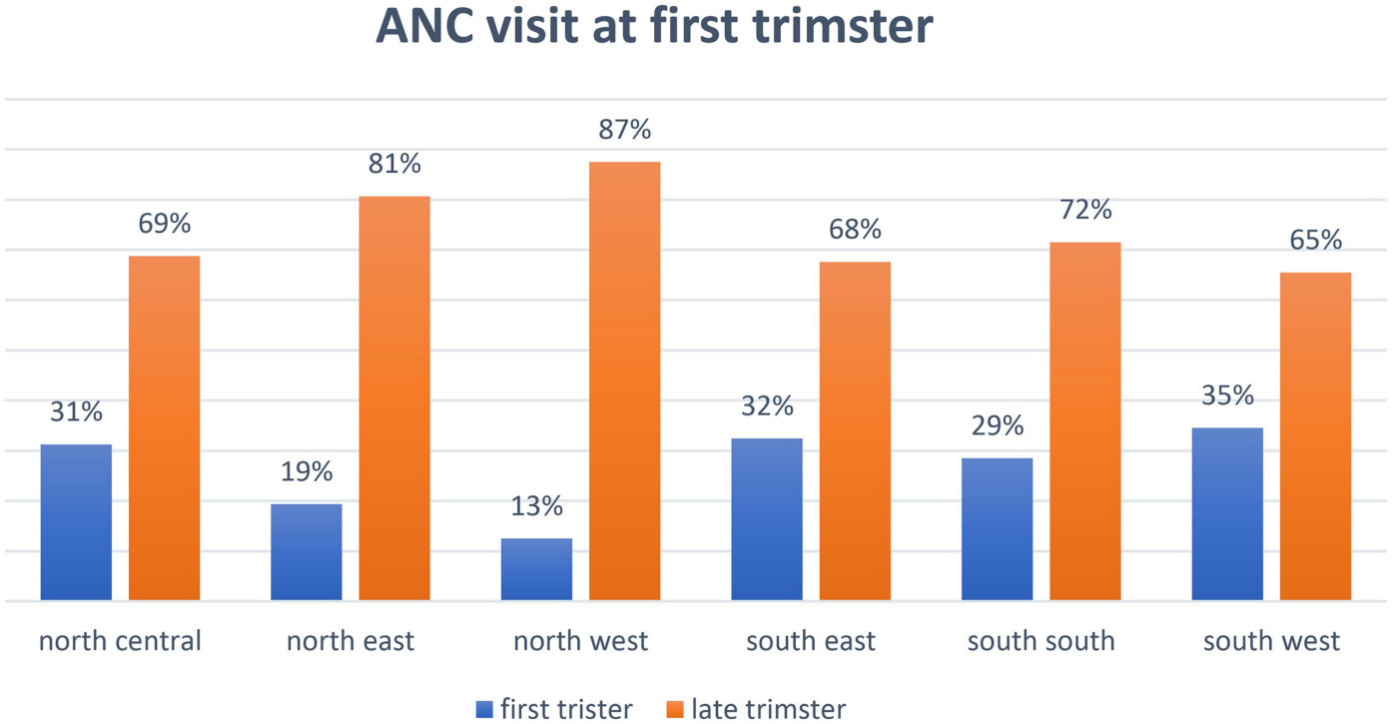
Proportion of first ANC visit at first trimester across the regions in Nigeria, 2018.

The overall prevalence of ANC visits during the first trimester across Nigeria with 24% commencing ANC at first trimester and 76% did not commence until later across Nigeria, revealing that only a minority of women commenced ANC early, with substantial regional variations. Figure 1 shows a map of Nigeria highlighting the regional disparities in the prevalence of first-trimester ANC visits. The South West region recorded the highest prevalence at 34.53%, while the North West region had the lowest at 12.51%. Figure 2 further depicts the proportion of first-trimester ANC visits across the regions, emphasizing significant regional disparities in ANC utilization.

### Bivariable association of sociodemographic factors with first ANC visit among reproductive-age women in Nigeria, 2018

Table 2 summarizes the bivariable analysis of sociodemographic factors associated with first-trimester ANC visits. The results indicate that women with higher education levels are significantly more likely to initiate ANC during the first trimester, with those having higher education showing an odds ratio (COR) of 2.32 (95% CI: 1.97–2.73) compared to women with no education. Wealthier women also had higher odds of early ANC visits, with the richest group having a COR of 2.88 (95% CI: 2.49–3.33). Employment status also showed a significant association, with employed women having higher odds (COR: 1.24, 95% CI: 1.12–1.38) of initiating early ANC visits. Regional analysis revealed that women in the North West were less likely to initiate early ANC compared to those in the North Central region (OR: 0.31, 95% CI: 0.27–0.36).

**Table 2:** Bivariable analysis of sociodemographic factors influencing timing of first ANC visit in Nigeria, 2018.

Variables	Category	Late trimester	Frist trimester	COR (95% C.I)	*P*-value
Maternal age	15–19	652 (80.64)	157 (19.36)	R.C	R.C
	20–24	2,369 (76.34)	734 (23.66)	1.17 (0.94–1.47)	0.021
	25–29	3,287 (75.12)	1,089 (24.88)	1.12 (0.90–1.39)	0.326
	30–34	2,823 (74.68)	957 (25.32)	1.06 (0.85–1.32)	0.605
	35–39	2,086 (75.44)	679 (24.56)	1.00 (0.80–1.25)	0.997
	40–44	936 (76.10)	294 (23.90)	1.03 (0.80–1.32)	0.804
	45–49	381 (79.51)	98 (20.49)	0.92 (0.67–1.26)	0.587
Educational level	No Education	4,684 (84.87)	835 (15.13)	R.C	R.C
	Primary	2,136 (77.63)	615 (22.37)	1.27 (1.10–1.48)	<0.001
	Secondary	4,525 (70.99)	1,849 (29.01)	1.58 (1.39–1.80)	<0.001
	Higher	1,189 (62.69)	708 (37.31)	2.32 (1.97–2.73)	<0.001
Religion	Catholic	1,079 (65.34)	572 (34.66)	R.C	R.C
	Other Christian	3,898 (68.00)	1,834 (32.00)	0.87 (0.76–1.00)	0.04
	Islam	7,491 (82.49)	1,590 (17.51)	0.53 (0.46–0.61)	<0.001
	Traditionalist	43 (83.43)	9 (16.57)	0.54 (0.22–1.30)	0.17
	Other	24 (91.07)	2 (8.93)	0.18 (0.08–0.43)	<0.001
Wealth index	Poorest	2,064 (83.98)	394 (16.02)	R.C	R.C
	Poorer	2,508 (79.74)	637 (20.26)	1.33 (1.15–1.55)	<0.001
	Middle	2,790 (77.75)	799 (22.25)	1.50 (1.30–1.74)	<0.001
	Richer	2,805 (76.14)	879 (23.86)	1.64 (1.41–1.91)	<0.001
	Richest	2,368 (64.57)	1,299 (35.43)	2.88 (2.49–3.33)	<0.001
Employment status	No	3,658 (80.70)	875 (19.30)	R.C	R.C
	Yes	8,877 (73.91)	3,133 (26.09)	1.24 (1.12–1.38)	<0.001
Births in Last 5 Years	1	6,450 (73.10)	2,373 (26.90)	R.C	R.C
	2	5,168 (78.81)	1,390 (21.19)	0.81 (0.74–0.89)	<0.001
	3	851 (79.04)	226 (20.96)	0.77 (0.65–0.92)	0.01
	4 +	66 (77.15)	19 (22.85)	0.85 (0.39–1.80)	0.69
Marital status	Never In Union	292 (71.79%)	115 (28.21%)	R.C	R.C
	Married	11,418 (76.10%)	3,587 (23.90%)	0.80 (0.57, 1.12)	0.19
	Living With Partner	381 (72.59%)	144 (27.41%)	0.96 (0.65, 1.41)	0.84
	Widowed	165 (77.91%)	47 (22.09%)	0.72 (0.44, 1.19)	0.20
	Divorced	162 (77.69%)	46 (22.31%)	0.73 (0.42, 1.28)	0.27
	Separated	116 (62.65%)	69 (37.35%)	1.52 (0.93, 2.46)	0.09
Residence	Urban	5,688 (72.79)	2,126 (27.21)	R.C	R.C
	Rural	6,847 (78.44)	1,882 (21.56)	0.99 (0.90–1.08)	0.80
Region	North Central	1,507 (68.76)	685 (31.24)	R.C	R.C
	North East	2,222 (80.64)	533 (19.36)	0.53 (0.46–0.61)	<0.001
	North West	4,257 (87.49)	609 (12.51)	0.31 (0.27–0.36)	<0.001
	South East	1,390 (67.58)	667 (32.42)	1.06 (0.92–1.21)	0.43
	South-South	1,177 (71.50)	469 (28.50)	0.88 (0.75–1.02)	0.10
	South West	1,981 (65.47)	1,045 (34.53)	1.16 (1.01–1.34)	0.04

### Random effect and model comparison

The analysis of the predictors of early antenatal care (ANC) visits was structured through three distinct multivariable multilevel logistic regression models. Model 1, the Null Model, served as the baseline to assess the variance attributable to regional differences without incorporating any predictors. This initial model revealed an intraclass correlation coefficient (ICC) of 6.1%, indicating a moderate level of clustering by region. The variance for this model was recorded at 0.214, with an Akaike Information Criterion (AIC) of 17,596.56 and a Bayesian Information Criterion (BIC) of 17,611.97. Building upon the Null Model, Model 2 incorporated individual-level predictors to examine their impact on the timing of early ANC visits. The improvements in model fit were evident, with a slightly reduced AIC of 17,592.75 and an increased BIC of 17,739.14 compared to Model 1. Model 3 demonstrated the best fit, with the lowest AIC and BIC values of 17,368.12 and 17,406.64, respectively, suggesting a significant improvement over the previous models. The variance decreased to 0.089, indicating a reduced random effect by region, and the ICC dropped to 2.6%. The sequential introduction of predictors across the models clearly demonstrated a pattern of decreasing ICC, AIC, and BIC, indicating an increasingly better fit of the models. Therefore, all interpretations and reports were made based on this model. Details of models along parameters indicated in Table 3.

**Table 3:** Random effect and model comparison for predictors of first trimester ANC visit in Nigeria, 2018.

Parameter	Null (model I)	Model II	Model II
Variance (Region)	0.21377342		0.08857188
AIC	17,596.562	17,592.75	17,368.116
BIC	17,611.972	17,739.143	17,406.641
loglikelihood	−8,796.2809	−8,777.3748	−8,679.058
ICC %	6.1%		2.6%

### Multivariable multilevel logistic regression analysis of factors associated with ANC visits at first-trimester

In the multivariable multilevel analysis results based on Model 3 (Table 4), which is the best-fitting model incorporating both community and individual-level factors, education, religion, wealth, and birth history emerged as significant predictors of early antenatal care (ANC) initiation. Women with higher education were nearly twice as likely to initiate ANC within the first trimester compared to those with no formal education (AOR = 1.98, 95% CI: 1.65–2.37). Women with secondary education also had significantly higher odds of early ANC initiation (AOR = 1.36, 95% CI: 1.14–1.62), whereas primary education was not a significant predictor (AOR = 1.12, 95% CI: 0.98–1.28).

**Table 4:** Multivariable multilevel logistic regression analysis for first trimester initiation of antenatal care among reproductive-age women in Nigeria, 2018.

Variables	Category	Model 1	Model 2	*p*-value	Model 3	*p*-value
			AOR (95% C.I)		AOR (95% C.I)	
Maternal age	15–19		R.C		R.C	0.34
	20–24		1.17 (0.94–1.47)	0.021	1.13 (0.88–1.46)	0.71
	25–29		1.12 (0.90–1.39)	0.326	1.05 (0.80–1.39)	0.99
	30–34		1.06 (0.85–1.32)	0.605	1.00 (0.73–1.37)	0.67
	35–39		1.00 (0.80–1.25)	0.997	0.94 (0.69–1.27)	0.91
	40–44		1.03 (0.80–1.32)	0.804	0.99 (0.77–1.26)	0.20
	45–49		0.92 (0.67–1.26)	0.587	0.89 (0.74–1.06)	0.34
Educational level	No Education		R.C		R.C	
	Primary		1.27 (1.10–1.48)**	<0.001	1.12 (0.98–1.28)	0.09
	Secondary		1.58 (1.39–1.80)**	<0.001	1.36 (1.14–1.62)**	<0.001
	Higher		2.32 (1.97–2.73)**	<0.001	1.98 (1.65–2.37)**	<0.001
Religion	Catholic		R.C		R.C	
	Other Christian		0.87 (0.76–1.00)*	0.042	0.87 (0.75–1.01)	0.07
	Islam		0.53 (0.46–0.61)**	<0.001	0.64 (0.47–0.87)**	0.01
	Traditionalist		0.54 (0.22–1.30)	0.169	0.65 (0.23–1.85)	0.42
	Other		0.18 (0.08–0.43)**	<0.001	0.21 (0.16–0.28)**	<0.001
Wealth index	Poorest		R.C		R.C	<0.001
	Poorer		1.33 (1.15–1.55)**	<0.001	1.33 (1.15–1.55)**	<0.001
	Middle		1.50 (1.30–1.74)**	<0.001	1.50 (1.30–1.74)**	<0.001
	Richer		1.64 (1.41–1.91)**	<0.001	1.64 (1.41–1.91)**	<0.001
	Richest		2.88 (2.49–3.33)**	<0.001	2.88 (2.49–3.33)**	<0.001
Employment status	No		R.C		R.C	
	Yes		1.24 (1.12–1.38)**	<0.001	1.14 (0.99–1.31)	0.07
Births in Last 5 Years	1		R.C		R.C	
	2		0.81 (0.74–0.89)**	<0.001	0.84 (0.78–0.89)**	<0.001
	3		0.77 (0.65–0.92)**	0.005	0.81 (0.69–0.97)*	0.02
	4 +		0.85 (0.39–1.80)	0.686	0.93 (0.33–2.61)	0.88
Residence	Urban		R.C		R.C	
	Rural		0.99 (0.90–1.08)	0.798	1.08 (0.89–1.32)	0.43

Statistical significance: *P < 0.05, **P < 0.001.

Religious affiliation influenced ANC timing. Compared to Catholic women, Muslim women were significantly less likely to initiate ANC early (AOR = 0.64, 95% CI: 0.47–0.87). Similarly, women in the “Other” religious category had a significantly lower likelihood of early ANC initiation (AOR = 0.21, 95% CI: 0.16–0.28). Economic status showed a strong association with early ANC initiation. Women in the richest wealth quintile were nearly three times more likely to initiate ANC early compared to those in the poorest quintile (AOR = 2.88, 95% CI: 2.49–3.33). A clear gradient was observed, with women in the middle-income group (AOR = 1.50, 95% CI: 1.30–1.74) and the poorer group (AOR = 1.33, 95% CI: 1.15–1.55) also showing higher odds of early ANC initiation.

Parity had a significant effect on early ANC initiation. Women who had three births in the last five years were less likely to initiate ANC early compared to those who had only one birth (AOR = 0.81, 95% CI: 0.69–0.97). Women with two births also had lower odds of early ANC (AOR = 0.84, 95% CI: 0.78–0.89). Employment status and place of residence were not significant predictors of early ANC initiation. Employed women had slightly higher odds of early ANC initiation, but the association was not statistically significant (AOR = 1.14, 95% CI: 0.99–1.31). Similarly, rural women had no significant difference in ANC initiation compared to urban women (AOR = 1.08, 95% CI: 0.89–1.32).

When community-level factors were included in the analysis, the influence of individual-level predictors such as education and wealth was slightly attenuated but remained significant, reinforcing the role of both individual and structural factors in determining ANC timing. Despite adjusting for multiple factors, significant regional disparities persisted, with the South West region maintaining the highest likelihood of first-trimester ANC initiation, while the North West region had the lowest rates even after controlling for other determinants.

## Discussion

This study provides an in-depth analysis of the sociodemographic determinants influencing early antenatal care (ANC) initiation among Nigerian women, highlighting significant regional, economic, and educational disparities. The findings indicate that only 24.0% of women initiated ANC within the first trimester, with notable differences across regions. The South West region recorded the highest prevalence (34.5%), while the North West region had the lowest (12.5%). These disparities reflect broader structural inequities in healthcare access and emphasize the need for region-specific maternal health interventions (25–27).

The results of this study align with previous findings on the importance of education in driving health-seeking behaviors. Women with higher education were nearly twice as likely to initiate ANC early compared to those with no education (AOR = 1.98, 95% CI: 1.65–2.37). This supports existing research from sub-Saharan Africa, where education is consistently linked to improved maternal healthcare utilization (28–31). Education enhances health literacy, promotes awareness of maternal health services, and reduces misconceptions surrounding ANC (20). These findings underscore the need for policies that prioritize female education, as it not only improves maternal healthcare access but also contributes to better overall health outcomes (29).

Economic status was another significant determinant, with wealthier women nearly three times more likely to initiate ANC early compared to their poorest counterparts (AOR = 2.88, 95% CI: 2.49–3.33). Similar patterns have been reported in other LMICs countries, where financial constraints remain a major barrier to ANC utilization (21, 32–34). Direct and indirect costs, such as transportation, consultation fees, and time off work, disproportionately affect low-income women, further limiting their access to timely maternal healthcare services (35). Economic empowerment programs, such as cash transfers and ANC subsidies, have been effective in improving ANC uptake in other low-resource settings and should be explored as a maternal health intervention strategy in Nigeria (36).

Religious affiliation also played a role in ANC timing, with Muslim women significantly less likely to initiate ANC early compared to Catholic women (AOR = 0.64, 95% CI: 0.47–0.87). Studies in other settings similarly found that cultural and religious norms influence perceptions of pregnancy care, affecting ANC utilization (37–39). Engaging religious leaders and faith-based organizations in maternal health education campaigns could improve ANC utilization, particularly in northern Nigeria, where religious and cultural beliefs play a strong role in shaping health-seeking behaviors (40).

Parity was another key determinant, as women with three births in the last five years were less likely to initiate ANC early compared to those with one birth (AOR = 0.81, 95% CI: 0.69–0.97). This finding aligns with studies in Ethiopia and Malawi, which suggest that high-parity women may deprioritize ANC due to previous pregnancy experiences, childcare responsibilities, or financial constraints (41, 42) (Basha, 2019). Strengthening community-based maternal health education programs could help reinforce the importance of ANC for women of all parity levels (43).

Interestingly, while employment status was significant in unadjusted models, it was not a significant predictor in the final adjusted model (AOR = 1.14, 95% CI: 0.99–1.31). This suggests that employment alone does not directly improve ANC access, but rather, its benefits are mediated by income, education, and workplace maternity policies. Studies in Gambia and other sub-Saharan African countries indicate that job security, employer-provided maternity benefits, and flexible work policies play a stronger role in ANC timing than employment itself (22, 44, 45).

Despite adjusting for individual and community-level factors, significant regional disparities in ANC initiation persisted. Women in the North West region remained the least likely to initiate ANC early, even after controlling for education, wealth, and employment status. This suggests that structural barriers, such as healthcare infrastructure deficiencies, provider shortages, and policy gaps, contribute to the low uptake of early ANC in northern Nigeria (46). Similar disparities have been observed in conflict-affected areas, where political instability, weak health systems, and sociocultural barriers hinder ANC utilization (14). Addressing these challenges requires multi-sectoral interventions, including health system strengthening, community outreach programs, and policy reforms tailored to the specific needs of each region (47).

The persistence of regional disparities despite controlling for individual factors suggests the need for policies that address systemic healthcare inequities. Investing in health infrastructure, improving ANC service accessibility, and promoting culturally sensitive health interventions could significantly enhance early ANC uptake across Nigeria (48, 49). Future research should explore barriers to ANC utilization at the healthcare system level, including provider attitudes, quality of ANC services, and regional policy differences. Additionally, qualitative studies examining women's experiences and perceptions of ANC services could provide deeper insights into context-specific challenges and guide more effective intervention strategies (49, 50).

## Conclusion

This study highlights significant regional and sociodemographic disparities in first-trimester antenatal care (ANC) initiation among Nigerian women, with higher education and wealth emerging as key predictors of early ANC uptake. Despite adjusting for individual and community-level factors, disparities persisted, particularly in the North West region, where ANC initiation rates remained the lowest. These findings underscore the need for targeted interventions, including improving female education, economic empowerment, and strengthening healthcare infrastructure, to enhance ANC utilization. Additionally, engaging religious and community leaders in maternal health campaigns may help address cultural barriers to healthcare access. Addressing systemic challenges through policy reforms, financial support mechanisms, and community-based outreach programs can promote equitable access to maternal healthcare services and ultimately improve maternal and neonatal health outcomes across Nigeria.

### Strength and limitation

This research utilizes a large, nationally representative sample from the 2018 Nigeria Demographic and Health Survey, ensuring broad generalizability of the findings. The use of multivariable multilevel logistic regression allows for the assessment of both individual and community-level factors, providing a comprehensive understanding of the determinants of early antenatal care (ANC) initiation. The cross-sectional design limits the ability to establish causality. Additionally, the reliance on self-reported data may introduce recall bias. The study may also not fully capture the experiences of marginalized populations, such as those in remote or conflict-affected areas, potentially underestimating regional disparities.

## Data Availability

The original contributions presented in the study are included in the article/Supplementary Material, further inquiries can be directed to the corresponding author.
